# Expanding the clinical spectrum of pediatric ataxia-telangiectasia: a case series of novel genetic variants, lupus vulgaris, and hyper-IgM phenotypes

**DOI:** 10.1186/s13023-025-03942-7

**Published:** 2025-10-03

**Authors:** Damla Baysal Bakır, Özge Atay, Halime Yağmur, Gizem Kabadayı, Mehmet Kocabey, Suna Asilsoy, Nevin Uzuner

**Affiliations:** 1 Faculty of Medicine, Department of Pediatric Allergy and Immunology, Dokuz Eylul University, Izmir, Turkey; 2 Faculty of Medicine, Department of Medical Genetics, Dokuz Eylul University, Izmir, Turkey

**Keywords:** Ataxia-telangiectasia, ATM, Hyper IgM, Lupus vulgaris, Malignancy

## Abstract

**Background:**

Ataxia-telangiectasia (A-T) is a rare autosomal recessive disorder caused by pathogenic *ATM* gene variants, characterised by progressive cerebellar ataxia, telangiectasia, immunodeficiency, and cancer predisposition. While its immunological and oncological complications are well-documented, clinical heterogeneity, particularly in cases with elevated IgM, poses diagnostic challenges.

**Methods:**

Following written informed consent, we retrospectively analysed four pediatric A-T patients followed in our clinic. Clinical, laboratory, and radiological data were reviewed, including immunoglobulin levels, vaccine antibody responses, lymphocyte subsets, and alpha-fetoprotein (AFP) levels. Diagnosis was established based on clinical and laboratory findings, supported by whole-exome sequencing (WES) and targeted *ATM* gene sequencing.

**Results:**

Our findings further support the association between the hyper-IgM phenotype and increased immune dysfunction in A-T. We report the first globally documented case of lupus vulgaris in an A-T patient and identify a previously unreported ATM variant in our country, expanding the disease spectrum. These findings highlight the need for further research on regional genetic variations and their clinical implications.

**Conclusion:**

This study highlights the importance of early diagnosis and genetic testing, particularly in atypical presentations. The recognition of novel infectious and autoimmune associations, along with novel variants, underscores the necessity of comprehensive, multidisciplinary follow-up and regional genetic screening efforts.

## Introduction

Ataxia-telangiectasia (A-T; MIM #208900) is a rare autosomal recessive disorder caused by pathogenic variants in the *ATM* (*Ataxia Telangiectasia*,* Mutated*) gene, located on chromosome 11q22.3 [1]. It is characterised by progressive cerebellar ataxia, oculocutaneous telangiectasia, immune deficiency, and a markedly increased risk of malignancy. The degree of residual ATM kinase activity determines the phenotypic severity, ranging from classical forms with biallelic loss-of-function mutations to milder, late-onset variants [2]. The estimated prevalence ranges from 1 in 40,000 to 1 in 300,000, with higher rates reported in populations with a high incidence of consanguinity [3]. Although precise national prevalence data are lacking, clinical series from Turkey have reported parental consanguinity in up to 88% of affected individuals, indicating a potentially increased regional burden of disease [4]. Classical A-T is commonly linked to nonsense or frameshift mutations, whereas variant forms are associated with missense or splice-site changes, supporting genotype–phenotype correlations [5–7].

A case of A-T is often normal during the early infantile period, with findings typically becoming apparent as gross motor skills develop, particularly during the sitting and walking stages. Those with the classic variant, especially, experience difficulty standing upright and walking unsteadily, and require support due to diffuse cerebellar degeneration. By age 5–6, telangiectasias become evident on the skin and conjunctiva, while oculomotor abnormalities and impaired visual fixation may also develop. Recurrent upper and lower respiratory tract infections are common and may result in frequent hospitalisations.

A-T is classified among chromosomal instability syndromes and DNA repair disorders. Increased radiosensitivity complicates the management of malignancies, which affect approximately 10–15% of children and up to 25% of individuals with the classical form [8]. The disease confers a high risk for lymphoid malignancies, less frequently, to solid tumours. Life expectancy is typically limited to 20–30 years [9].

Immune dysfunction is a key feature of A-T and significantly contributes to morbidity and mortality. In possible and probable cases, the diagnosis is established based on the European Society for Immunodeficiencies (ESID) criteria, which incorporate laboratory and clinical findings alongside progressive cerebellar ataxia and evidence of increased radiation-induced chromosomal breakage in cultured cells [10]. Combined immunodeficiency, affecting both humoral and cellular compartments, is observed in approximately two-thirds of patients, resulting in recurrent sinopulmonary infections, autoimmunity, and chronic inflammation [11]. Pediatric cases often exhibit lymphopenia, low levels of IgG, IgA, IgE or IgG subclasses, and impaired vaccine responses. A minority may present with elevated IgM, which can lead to a misdiagnosis of hyper-IgM syndrome [12, 13]. These immunological abnormalities are associated with increased risks of bronchiectasis, autoimmune complications, and malignancy, contributing to poor prognosis.

Another notable finding in A-T patients is elevated serum alpha-fetoprotein (AFP) levels. Increased AFP levels are also observed in certain forms of severe combined immunodeficiency (e.g., ADA deficiency) and in oculomotor apraxia type 2, which shares a similar clinical course [14, 15]. However, the underlying mechanism remains poorly understood.

This study describes the clinical and immunological features of four pediatric A-T cases managed in our clinic. It expands the clinical spectrum of A-T by reporting elevated IgM levels, genotype–phenotype correlations, and, for the first time globally, an association with lupus vulgaris.

## Methods

Clinical, laboratory, and radiological data of four pediatric patients diagnosed with A-T and followed in our pediatric immunology clinic were retrospectively analysed. The evaluation included medical history, physical and neurological examinations, and immunological investigations. Laboratory tests comprised complete blood count (CBC), serum immunoglobulins, vaccine responses, lymphocyte subsets, and AFP levels. Imaging studies (HRCT, cranial/thoracic MRI and PET) were reviewed for pulmonary, neurodegenerative and oncological involvement.

Variant analysis and classification were conducted according to ACMG guidelines [16] and cross-referenced with ClinVar, HGMD, CentoMD, and gnomAD databases. Associated clinical features, including autoimmunity, malignancies, and susceptibility to infections, were evaluated in relation to previously reported cases in the literature.

## Results

### Patient 1

An 8-year-old male was initially evaluated at another centre at the age of 3 due to recurrent otitis media, occurring 3–4 times per year. Laboratory investigations revealed severe anaemia (haemoglobin: 6 mg/dL) with a positive direct Coombs test, for which he received erythrocyte transfusion. Notably, his serum IgM level was markedly elevated (2620 mg/dL), prompting referral to our clinic with a primary diagnosis of primary immunodeficiency.

The patient had a history of two hospitalisations for pneumonia, treated by systemic antibiotics. Parental consanguinity (first cousins) was noted. Two siblings had died in infancy—one from neonatal sepsis due to omphalitis and the other from diarrhoea at 3 months of age.

On physical examination, his weight was below the 3rd percentile. Bilateral pulmonary rales were present, and the spleen was palpable 3 cm below the costal margin. No dysmorphic features or other systemic abnormalities were detected.

#### Immunology

CBC revealed no cytopenia. Immunoglobulin levels were markedly altered: IgG < 75 mg/dL (<-2 SD), IgA < 10 mg/dL (<-2 SD), and IgM elevated at 2590 mg/dL ( > + 2 SD). Peripheral lymphocyte subset analysis (PLSA) showed normal T and NK cell counts and distribution, while CD19^+^ B cells were reduced to 3.5% (reference: 14–44%) despite a normal absolute count. Serum AFP was elevated at 32 ng/ml (reference: 0-10ng/ml). No serologic response was detected following rubella vaccination.

Further evaluation for X-linked hyper IgM syndrome revealed normal expression of the CD40 marker, CD40 ligand, and replication function. No pathogenic variants were detected in the *CD40L* gene by DNA sequencing. The patient was initiated on immunoglobulin replacement therapy (IgRT) (0.6 g/kg every four weeks) and Trimethoprim-sulfamethoxazole (TMP-SMX) prophylaxis. At age 4, he developed lymphopenia (absolute lymphocyte count (ALC): 900 × 10^3^/µL), with elevated IgM and a decline in CD19^+^ B cells (1.5%). The patient was referred to pediatric genetics to investigate alternative molecular causes potentially associated with hyper-IgM phenotype.

#### Comorbidities

**Fig. 1:**
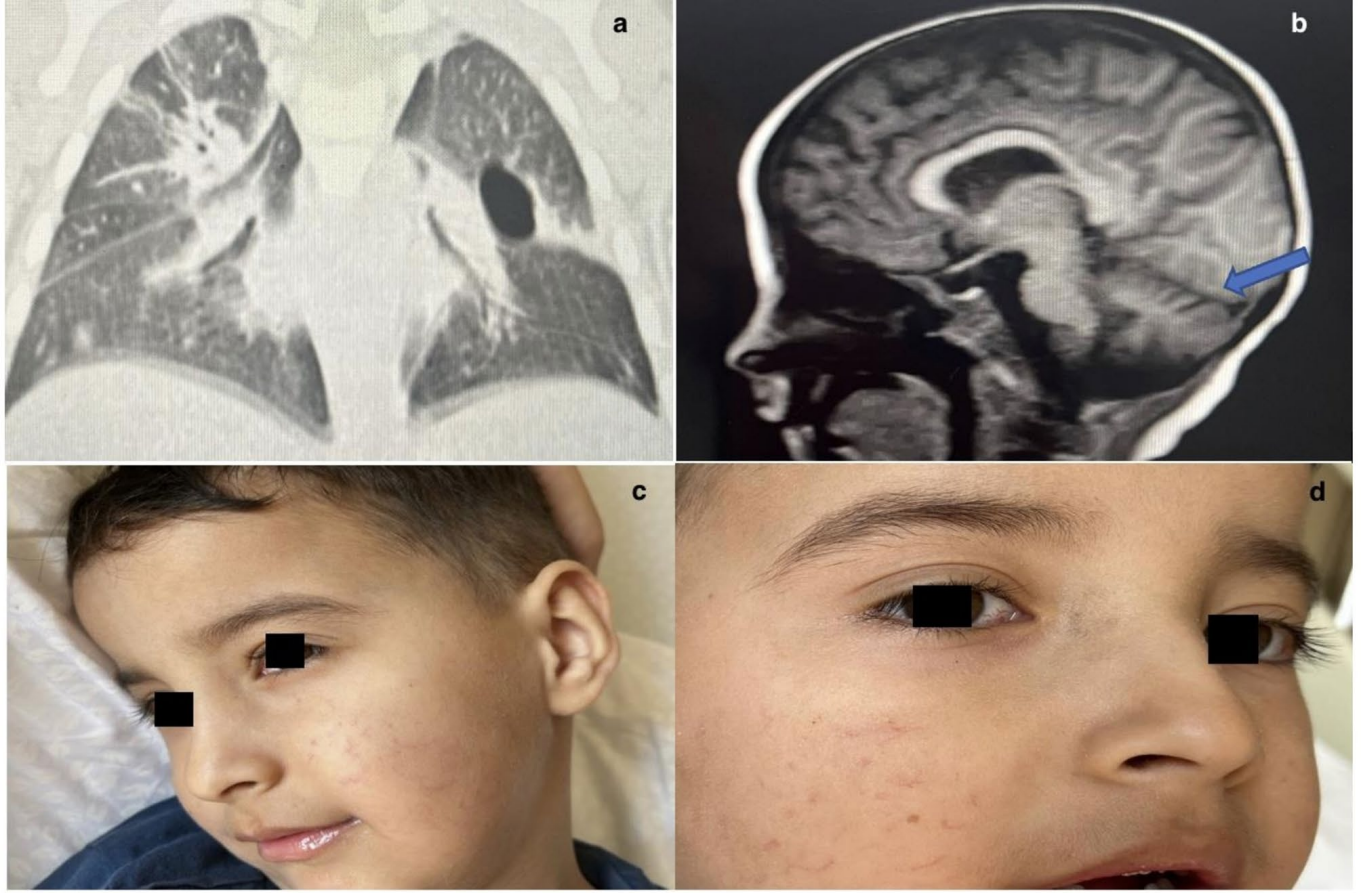
Imaging and examination findings of Patient 1. (**a**) HRCT image reveals a cystic appearance in the left lung, bilateral consolidation, and widespread atelectasis. (**b**) T1-weighted cranial MRI demonstrating cerebellar atrophy. (**c**, **d**) Telangiectasias visible on the cheeks and bulbar conjunctiva

Due to the patient’s ongoing respiratory symptoms, HRCT was performed, revealing pneumonic consolidations in the right lung, along with cystic changes, atelectasis, and air trapping in the left lung (Fig. 1a). These radiological abnormalities were correlated with clinical history and interpreted as sequelae of recurrent pulmonary infections.

#### Neurology

At age 5, following a two-year gap in follow-up, the patient re-presented with a whole exome sequencing (WES) result, confirming a diagnosis consistent with A-T. On examination, he exhibited telangiectasias on the cheeks and bulbar conjunctiva (Fig. 1c, d). Neurological examination revealed gait imbalance, oculomotor apraxia, and cognitive delay. Cranial MRI revealed cerebellar atrophy, consistent with the patient’s neurological findings (Fig. 1b).

#### Genetics

WES was performed at the Dokuz Eylul University Genetic Diseases Evaluation Center, Turkey. To generate exome capture libraries, the Twist Exome 2.0 kit (Twist Bioscience, USA) enriched 36.5 Mb of the human consensus coding sequence (targeting > 98% of RefSeq and Gencode v28 regions obtained from the human genome) according to the manufacturer’s protocols. The generated library was sequenced on the MGI DNBSEQ-G400 NGS platform to obtain a minimum read depth of 20x for > 98% of the targeted bases.

As a result of sequencing, raw data is obtained in FASTQ format. Sequence alignment, variant calling and annotation were performed on FASTQ data using the Franklin by Genoox program. All disease-causing variants reported in the HGMD^®^, ClinVar, and CentoMD^®^ databases and variants with a minor allele frequency (MAF) below 1% in the gnomAD database are considered. The search for relevant variants focused on the coding exons and surrounding +/- 20 intronic bases. In addition, family history and clinical information provided were used to evaluate identified variants based on their pathogenicity and disease causation. All variants were classified according to ACMG guidelines.

**Fig. 2:**
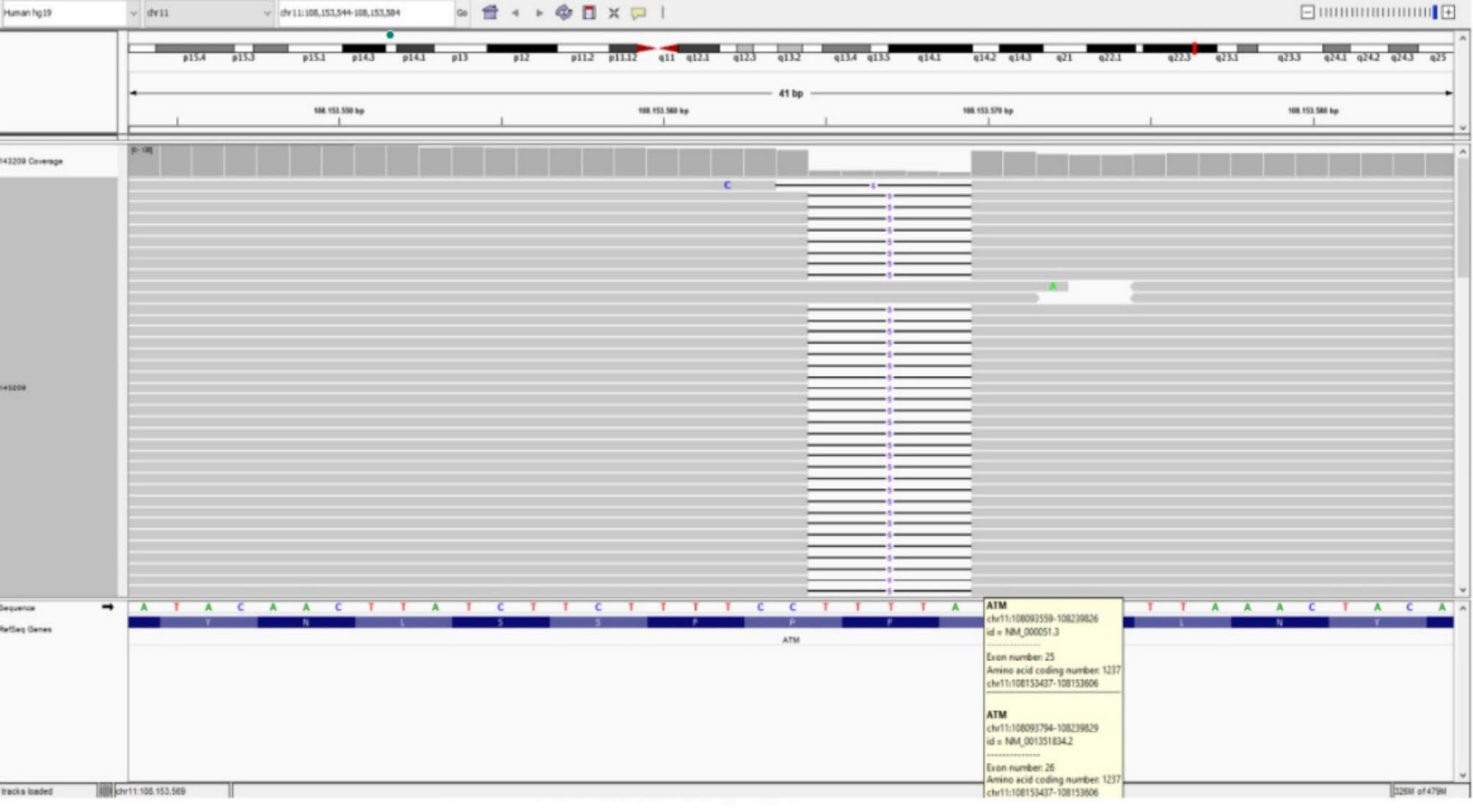
Integrative Genome Viewer (IGV) based analysis revealing the variant c.3712_3716delTTATT/p.Leu1238fs in Patient 1

Genetic sequencing revealed a homozygous c.3712_3716delTTATT (p.Leu1238Lysfs*6) frameshift variant in exon 24 of the *ATM* gene, resulting in a premature stop codon after six amino acids. This variant was classified as pathogenic based on ACMG criteria: PVS1 (null variant in a gene where loss of function is a known mechanism of disease), PM2 (absent from GnomAD v2), and PM3 (previously reported in trans with pathogenic variants) [17]. The mutation is associated with increased radiosensitivity and heightened risk for malignancies, including pancreatic adenocarcinoma and breast cancer. While previously reported in homozygous or compound heterozygous individuals, this is the first known case from Turkey (Fig. 2).

Following variant identification, parental carrier testing and extended family screening were initiated. Genetic counselling was provided for reproductive planning and risk assessment. Given the consanguineous background, the two deceased siblings were presumed to have shared the same mutation. The patient is monitored monthly, and no additional complications have been observed to date over a follow-up period of 24 months.

### Patient 2

#### Neurology

**Fig. 3:**
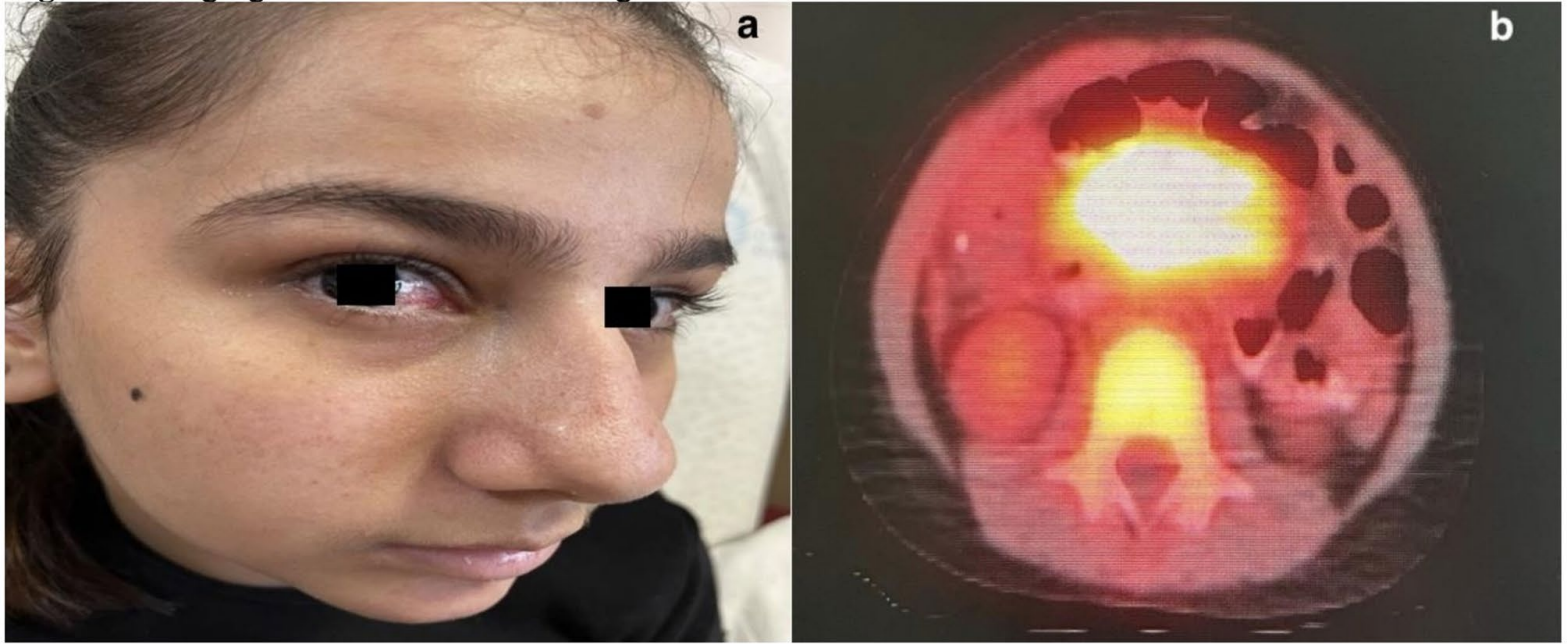
Clinical and imaging findings for Patient 2. (**a**) Telangiectasias in the bulbar conjunctiva. (**b**) FDG-PET showing pathological uptake in abdominal lymph nodes and spleen, consistent with diffuse large B-cell lymphoma

A 13-year-old girl was referred to our clinic at 7 months of age with ocular telangiectasias (Fig. 3a), poor head control, axial hypotonia, and sitting imbalance. The parents, who were first-degree cousins, also reported observing involuntary eye movements in the child. Family history revealed that her 16-year-old brother had previously died from A-T.

On examination, she exhibited hypotonia, reduced lower limb reflexes, extremities, and nystagmus. Growth and developmental delays were present, with weight and height below − 2 SD.

#### Immunology

Laboratory evaluation revealed lymphopenia (ALC: 900 × 10³/µL). Immunoglobulin levels were as follows: IgA < 10 mg/dL (< -2 SD), IgG 457 mg/dL (-1 to -2 SD), IgM 65 mg/dL (-1 to -2 SD), and IgE < 1 mg/dL. No antibody response was detected to rubella or hepatitis B vaccines. PLSA showed T cells, B cells, NK cells, and HLA-DR expression within normal limits despite reduced percentages. Serum AFP was elevated at 65.86 ng/mL (reference: 0–20 ng/mL).

Given the clinical presentation and positive family history, ATM genetic testing was planned at age one; however, the patient was lost to follow-up before completion.

#### Comorbidities

At age 3, she was diagnosed with oligoarticular juvenile idiopathic arthritis (JIA) based on bilateral knee swelling, pain, and morning stiffness. Methotrexate therapy was initiated by the pediatric rheumatology team.

At age 6, she was re-evaluated in our clinic following a systemic varicella infection requiring intravenous acyclovir and an increased frequency of sinopulmonary infections. Given the progressive clinical deterioration and immunological findings, *ATM* gene sequencing was performed, confirming the diagnosis of A-T. IgRT (0.6 g/kg every four weeks) and TMP-SMX were subsequently initiated.

At age 9, the patient presented to the emergency department with acute abdominal pain. Abdominal ultrasonography identified a 5 cm intussusception at the hepatic flexure, necessitating surgical intervention. Histopathology of a 1 cm mesenteric lymph node revealed diffuse large B-cell lymphoma (DLBCL). PET imaging showed pathological F-18 fluorodeoxyglucose (FDG) uptake in lymph nodes along the abdominal aorta and spleen (Fig. 3b). Additionally, there was a diffuse heterogeneous tissue and widespread symmetrical uptake in the bones and bone marrow. Given the aggressive disease course, a reduced-intensity R-CHOP regimen (including rituximab, cyclophosphamide, doxorubicin hydrochloride, vincristine, and prednisone) was initiated by the pediatric haematology team following multidisciplinary consultation. The protocol was adapted in light of the patient’s underlying diagnosis of A-T and associated radiosensitivity, and the patient was closely monitored throughout the treatment.

She remains under multidisciplinary follow-up and is currently receiving methotrexate with folic acid, IgRT, antibiotic prophylaxis, and respiratory physiotherapy.

### Patient 3

An 11-year-old girl was referred to our clinic at age 2 with a preliminary diagnosis of immunodeficiency due to recurrent upper respiratory tract infections (URTI) and pneumonia episodes, requiring hospitalisation twice a year.

#### Neurology

The patient was born to second-degree consanguineous parents, with a family history of A-T in two cousins. By age 1, delayed gross motor milestones—including difficulty sitting and gait instability— were noted. On examination, she exhibited minimal telangiectasias on the bulbar conjunctiva, delayed motor development, and decreased deep tendon reflexes (DTR).

#### Immunology

Laboratory evaluation revealed lymphopenia ALC: 1400 × 10³/µL). Immunoglobulin levels showed IgG < 75 mg/dL (< -2 SD), IgA < 10 mg/dL (< -2 SD), and IgE < 1 mg/dL (< -2 SD), and elevated IgM at 171 mg/dL ( > + 2 SD). PLSA showed reduced CD8^+^ T cells, while CD4^+^ T cells, B cells, NK cells, and HLA-DR expression were within normal limits. Protective antibody responses to rubella and hepatitis B vaccines were observed. The patient was initiated on IgRT (0.6 g/kg every 4 weeks) and TMP-SMX prophylaxis. During follow-up, IgM levels increased to 1270 mg/dL, and AFP was elevated at 90 ng/mL (reference: 0–20 ng/mL).

#### Comorbidities

At age 10, the patient presented with persistent cough and bilateral crepitant rales. Thoracic MRI revealed widespread right basal consolidation, hilar and mediastinal lymphadenopathy, and a 1.5 × 2 cm hyperintense lesion in liver segment VI on T2-weighted imaging. These findings were interpreted as non-specific and not indicative of malignancy.

Tuberculosis screening revealed a 5 mm PPD induration, negative interferon-gamma release assay and negative PCR and cultures for acid-fast bacilli in sputum and gastric fluid samples.

**Fig. 4:**
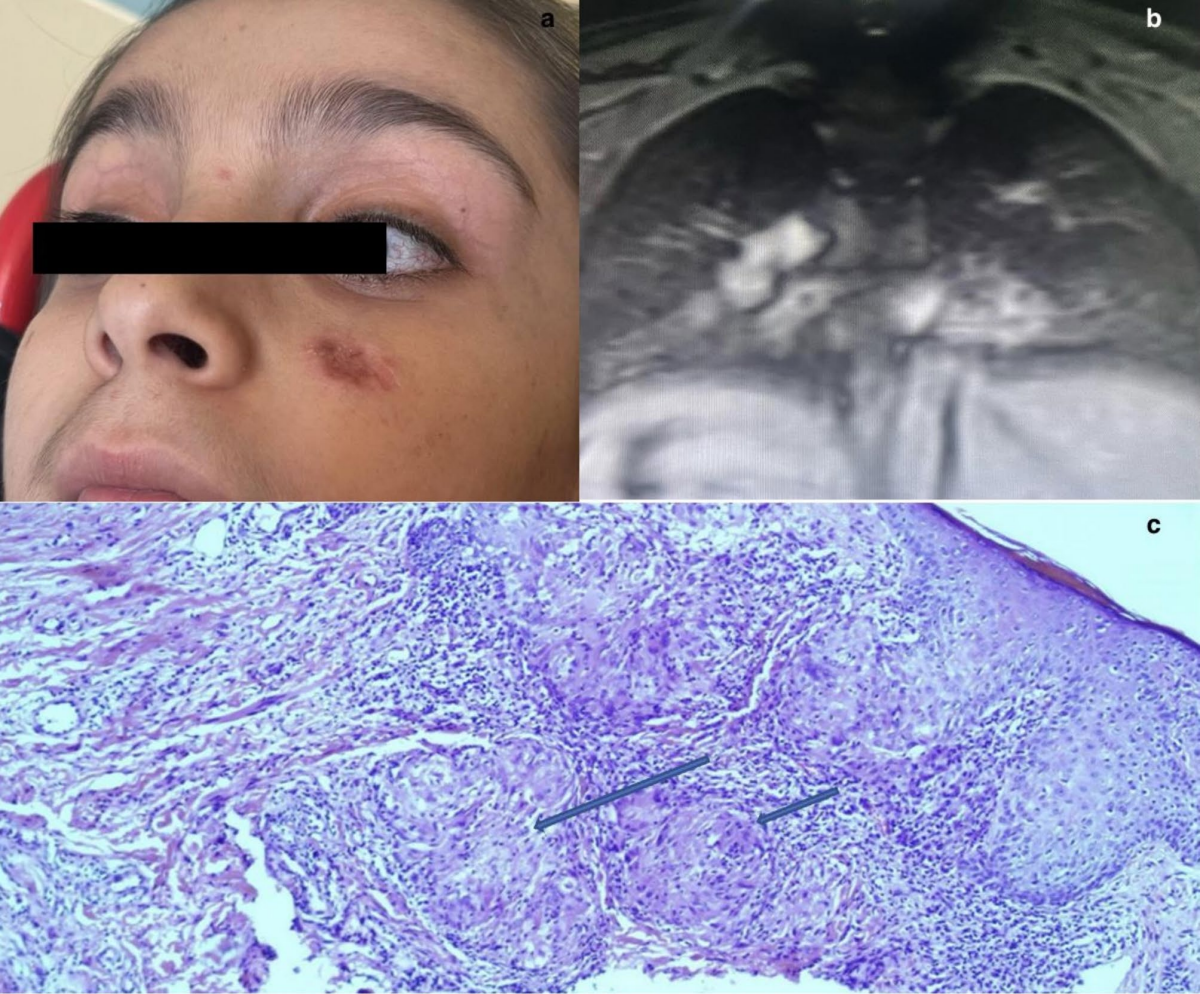
Clinical, radiological, and pathological findings of Patient 3. (**a**) Lupus vulgaris lesion on the left infraorbital region and conjunctival telangiectasias. (**b**) Thoracic MRI showing widespread hilar and mediastinal lymphadenopathy with parenchymal consolidation. (**c**) Incisional biopsy, stained with hematoxylin and eosin, demonstrating the presence of Langhans-type giant cells and caseous necrotic granulomas within the dermis

Concurrently, a 2 × 1 cm growing lesion with a scarred, hyperemic surface was observed on the left infraorbital-buccal region (Fig. 4a, b). Initial incisional biopsy suggested chronic cutaneous granuloma. Due to progressive enlargement of the lesion during follow-up, the biopsy was re-evaluated, revealing granulomatous inflammation with caseous necrosis and Langhans-type giant cells. Although PCR testing for Mycobacterium tuberculosis was negative, Ziehl-Neelsen staining demonstrated acid-fast bacilli, confirming the diagnosis of lupus vulgaris (Fig. 4c).

Although endobronchial ultrasound-guided lymph node biopsy was negative for *M. tuberculosis*, clinical and histopathological findings supported extrapulmonary TB. Standard anti-tuberculosis therapy (isoniazid, rifampicin, ethambutol, pyrazinamide, pyridoxine) was initiated.

Abdominal MRI at the third month of therapy demonstrated a mild reduction in thoracic lymphadenopathy, while no hepatic lesions suggestive of active tuberculosis were observed. Thus, the diagnosis of extrapulmonary tuberculosis remained limited to cutaneous involvement. Following the initial three-month intensive phase with the four-drug regimen, continuation therapy with isoniazid and rifampicin was planned for at least six additional months, under the coordination of the pediatric infectious disease clinic.

The patient remains under multidisciplinary follow-up, including immunology and pediatric infectious disease clinics, pediatric oncology for malignancy surveillance, and neurology and physical therapy services for the management of ataxia and progressive motor impairment.

#### Genetics

**Fig. 5:**
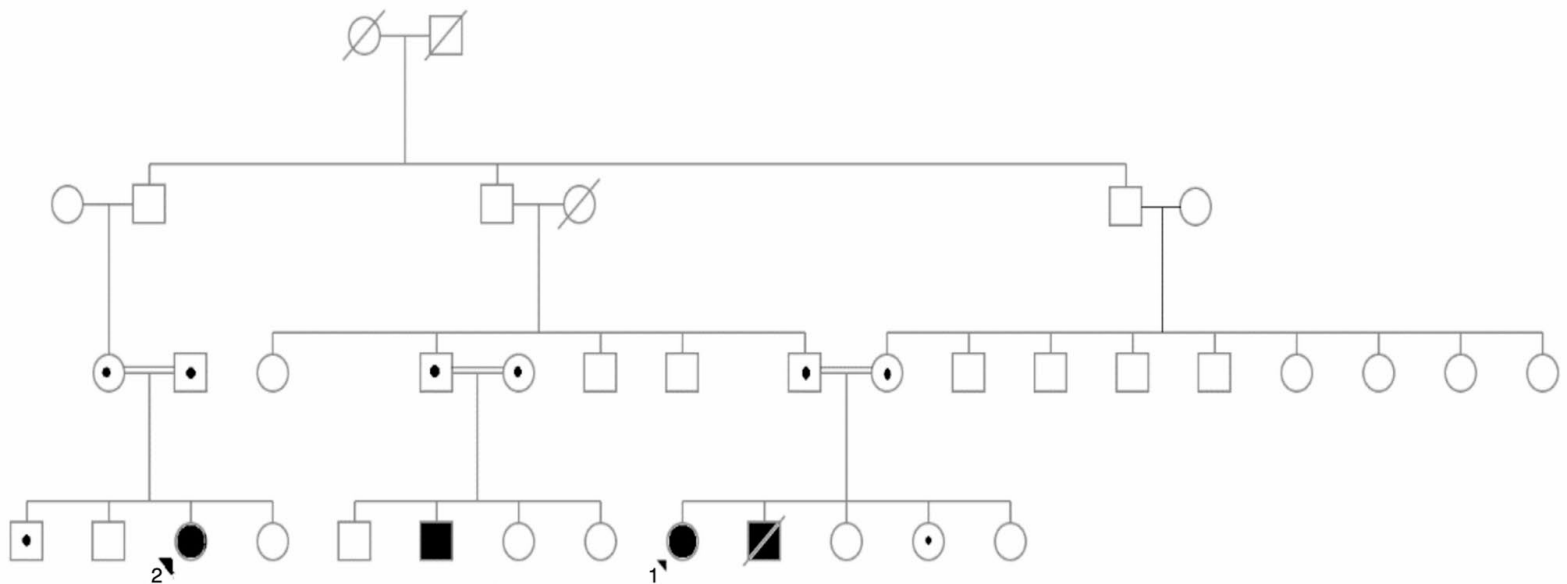
Pedigree chart depicting the family structure, illustrating consanguineous marriages and the affected children. 1: corresponds to Patient 2, and 2: corresponds to Patient 3

As depicted in the pedigree (Fig. 5), Patients 2 and 3 are paternal first cousins, each born to consanguineous parents. In both patients, a homozygous c.6047 A > G (p.D2016G) variant was detected in exon 41 of the *ATM* gene. This variant is a rare missense mutation not found in GnomAD v2 (PM2) and has been previously reported in trans position with other pathogenic mutations in A-T cases (PM3) [18]. In silico scores strongly predict that it is pathogenic (PP3 > M). Functional studies have been performed on this mutation previously, revealing only trace amounts of the protein in immunoblotting and an increased sensitivity to radiation in cell lines (PS3) [19]. With these criteria, we classified this variant as pathogenic.

**Fig. 6:**
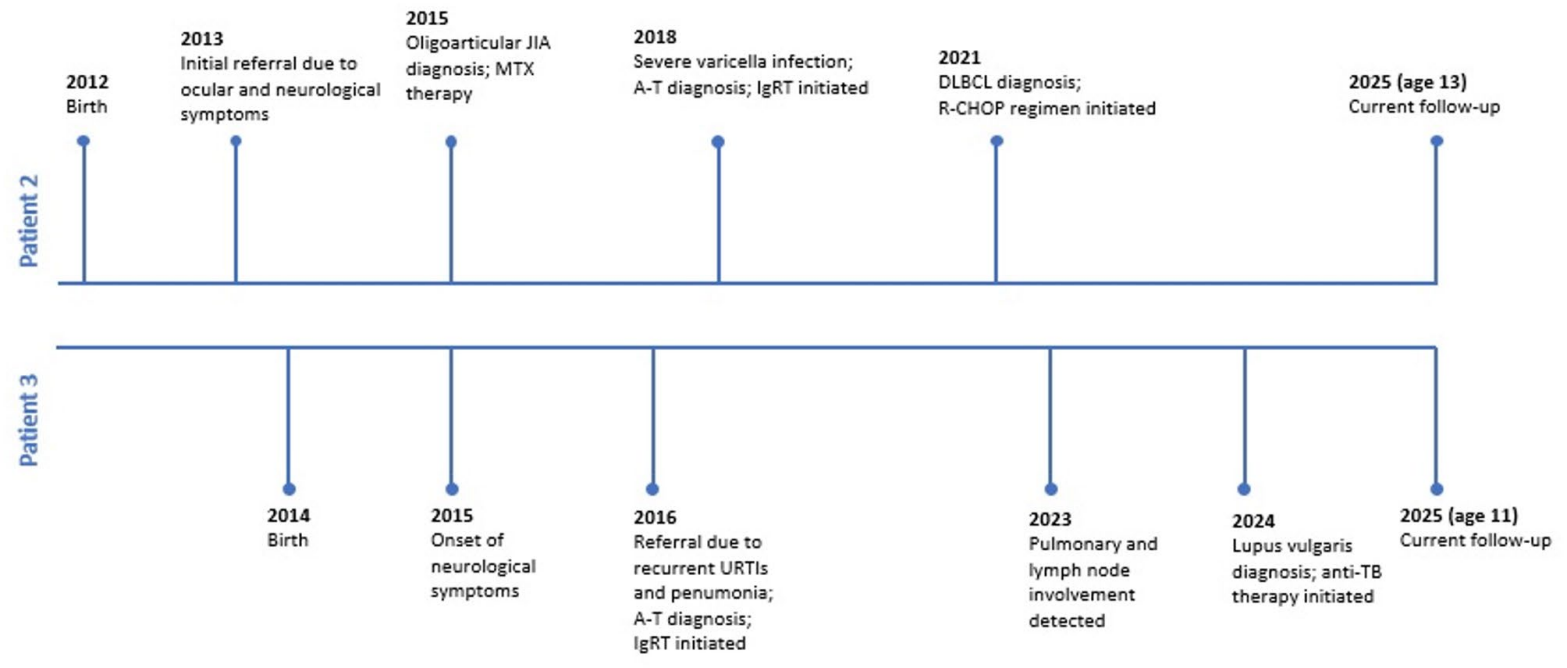
Clinical timelines of Patient 2 and Patient 3, showing chronological progression of major clinical events including disease onset, autoimmune manifestations, infections, and diagnosis of malignancy. **Abbreviations**: A-T: Ataxia-Telangiectasia; DLBCL: Diffuse Large B-Cell Lymphoma; IgRT: Immunoglobulin Replacement Therapy; JIA: Juvenile Idiopathic Arthritis; MTX: Methotrexate; TB: Tuberculosis; TMP-SMX: Trimethoprim-Sulfamethoxazole; URTI: Upper Respiratory Tract Infection

This variant may represent a recurrent *ATM* variant in the Turkish population since it has been observed in many families. Moreover, its heterozygous form has been associated with moderate breast cancer risk in Turkish cohorts [20]. Following the identification of the genetic variant and carrier cases through sequence analysis, genetic counselling was provided to the patients’ families. The clinical timelines of Patients 2 and 3 are presented in Fig. 6.

### Patient 4

#### Neurology

A 5-year-old girl was initially evaluated at an external centre due to progressive gait ataxia. Cranial MRI was reported as normal. Subsequently, a next-generation sequencing (NGS) panel identified a homozygous pathogenic variant in the *ATM* gene, confirming the diagnosis of A-T. The patient was referred to the pediatric neurology department of our institution. On examination, she exhibited pronounced ataxia, impaired tandem gait, persistent drooling, and an open-mouth posture. Serum AFP was markedly elevated at 352 ng/mL (reference: 0–5 ng/mL). Despite the genetic confirmation, the neurology team noted a discordance between the patient’s marked neurological symptoms and the unremarkable cranial MRI. Longitudinal follow-up was initiated, and the patient was referred to our clinic for comprehensive immunological evaluation.

#### Comorbidities

The patient’s history included progressively worsening ocular redness since age two, frequent febrile URTI (4–5 times per year), often requiring systemic antibiotic therapy. She was born to consanguineous parents with no family history of immunodeficiency. Two siblings were reported to be healthy.

On physical examination, her height was below the 3rd percentile, with telangiectasias observed on the bilateral bulbar conjunctiva and cheeks.

#### Immunology

Laboratory evaluation revealed a borderline ALC (1500 × 10³/µL), IgG level of 1385 mg/dL ( > + 2 SD), IgM at 166 mg/dL (+ 1 to + 2 SDS), and IgA < 10 mg/dL (<-2 SDS). Vaccine-specific responses to rubella and hepatitis B were presented. PLSA revealed normal counts for CD3^+^, CD4^+^, CD8^+^, CD19^+^, and CD16^+^/56^+^ cells. Lymphocyte proliferation assays were not performed due to technical limitations. Nonetheless, the preserved vaccine-specific antibody responses and normal distribution of lymphocyte subsets were suggestive of partially retained adaptive immune function.

The patient was discharged on TMP-SMX prophylaxis and initiated on IgRT. Follow-up was coordinated with pediatric immunology, neurology and physical rehabilitation services in her home province.

#### Genetics

A homozygous c.27del (p.Ile10Serfs*6) variant in exon 2 of the *ATM* gene was identified. Similar to the variant in Patient 1, this frameshift mutation leads to a premature stop codon formation after six amino acids. The variant is predicted to cause a null phenotype (PVS1). It was previously reported as pathogenic in the ClinVar database (PP5) and is absent from gnomAD v2 (PM2). Accordingly, we classified this variant as pathogenic.

This frameshift alteration was previously reported as a novel variant in a Turkish study [21]. Unfortunately, no information was available regarding any potential familial relationship with our patient. The study by Işık et al. discussed the clinical utility of NGS in a genetically diverse cohort with limited clinical details. Therefore, this case provides valuable clinical insight and complements previously reported data.

## Discussion

The *ATM* gene plays a central role in maintaining genomic integrity. Most pathogenic variants are truncating mutations that lead to the production of unstable or non-functional proteins. Complete loss of ATM kinase activity is associated with the classic, severe A-T phenotype, whereas residual activity in variant cases results in a milder, slower-progressing clinical course.

Our study illustrates the clinical heterogeneity observed among four pediatric A-T cases, including variability in presenting symptoms, immunological profiles, disease progression, and complications, which may reflect underlying genetic differences. These findings support previous literature emphasising genotype–phenotype correlations and highlight the role of specific ATM variants in determining disease severity and clinical course [22]. Notably, the variants identified in our study have been previously associated with the classical A-T phenotype, marked by recurrent infections, neurodegeneration, and increased malignancy risk. A comparative summary with previously reported cases is provided in Table 1, highlighting both shared and unique features.

**Table 1:** Comparison of clinical and genetic characteristics of our pediatric A-T patients and previously reported cases sharing the same ATM variants

**Variant: ATM: c.3712_3716delTTATT/** ***p*** **.Leu1238fs**						
Patient	Sex	Age at onset	Clinical findings	Laboratory findings	Comorbidities	
Present patient 1	M	3	Telangiectasias, frequent RTI, ataxia, growth retardation, splenomegaly, lung rales, splenomegaly	↓IgG/IgA, ↑IgM, ↓CD19 + B cells, ↓vaccine response, ↑AFP	Autoimmune hemolytic anemia, chronic lung disease	
Ref [17]	M	24	Ataxia, dystonia, cognitive alteration, cerebellar atrophy	Mild ↑AFP (9.23 µg/L; ref < 7 µg/L)	Na	
Ref [27]	M	8	Conjunctival telangiectasias, oculomotor apraxia, nystagmus, dysarthria, gait instability, cerebellar atrophy, axonal sensorimotor neuropathy, recurrent sinopulmonary and soft tissue infections	↑AFP, ↓IgA	Na	
Ref [28]	Na	71	Severe and rapidly progressive clinical course	Na	CLL	
**Variant: ATM: c.6047 A > G/p.D2016G**						
Patient	Sex	Age at onset	Clinical findings	Laboratory findings	Comorbidities	
Present patient 2	F	7 m	Nystagmus, difficulty sitting, poor head control, hypotonia, diminished DTRs	Lymphopenia, ↓IgG/IgA/IgM/IgE, ↓B and T cell subsets, ↓vaccine response, ↑AFP	Oligoarticular JIA, diffuse large B-cell lymphoma	
Present patient 3	F	2	Frequent RTI, diminished DTRs, delays in gross motor skills, telangiectasias	Lymphopenia, ↓IgG/IgA/IgE, ↑IgM, ↓CD8 + T cells, ↓vaccine response, ↑AFP	Lupus vulgaris	
Ref [29]	M	2	Difficulty walking, involuntary movements in the extremities and eyes, dystonia, myoclonus, resting tremor	↓IgG/IgA/IgM, ↓vaccine response	Type 1 DM	
Ref [22]	F	2	Telangiectasias, oculomotor apraxia, difficulty walking, frequent infections	↑AFP	Na	
**Variant: ATM: c.27del/p.Ile10SerfsTer6**						
Patient	Sex	Age at onset	Clinical findings	Laboratory findings	Comorbidities	
Present patient 4	F	16 m	Telangiectasias, difficulty walking, growth retardation, ataxia	↓IgA ↑AFP	Absent	
Ref [21]	F/M (siblings)	Na	Ataxia	Na	Dandy walker malformation and cerebellar atrophy	

Abbreviations: A-T: Ataxia-Telangiectasia; AFP: Alpha-Fetoprotein; Ig: Immunoglobulin; DTR: Deep Tendon Reflexes; JIA: Juvenile Idiopathic Arthritis; DLBCL: Diffuse Large B-Cell Lymphoma; DM: Diabetes Mellitus; RTI: Respiratory system infections; Ref: Reference; Na: Not available

*Variant classifications are based on ACMG criteria and previously published literature where available.*

As seen in two of our cases, the diagnosis of A-T was delayed. This delay was attributed to poor follow-up adherence, the late appearance of telangiectasia, and the milder course observed in variant forms. In such cases, clinical and laboratory features may not indicate immunodeficiency. Some may even resemble disorders such as hyper-IgM syndrome [23]. The phenotypic overlap contributed to the diagnostic delay in Patient 1, who was initially misclassified until WES confirmed the diagnosis. This underscores the diagnostic utility of WES in phenotypically atypical cases, particularly when classical hallmarks are absent and immunological profiles are inconclusive.

A clinically relevant observation in our cohort was the presence of autoimmune manifestations in two patients. One had a prior history of autoimmune hemolytic anaemia, while another developed juvenile idiopathic arthritis (JIA) during follow-up, requiring methotrexate under pediatric rheumatology care. Recent pediatric studies have highlighted the association between A-T and JIA, often presenting with joint swelling, pain, and signs of immune dysregulation [24].

This dysregulation involves varying degrees of humoral and cellular immune dysfunction, autoimmune cytopenias, and vitiligo. It is thought to result from ATM mutations affecting class-switch recombination and lymphocyte maturation. Defective DNA double-strand break repair impairs isotype switching and V(D)J recombination, reducing lymphocyte diversity and increasing autoimmunity [25]. Notably, Patient 2 developed both juvenile idiopathic arthritis and hematologic malignancy, suggesting a possible additive role of immune dysregulation in lymphomagenesis [26]. These findings underscore the need for close monitoring in A-T patients with concomitant immune dysregulation clinics.

Another point to be emphasised is the impact of the immunological manifestations of patients on the clinical phenotype. Most A-T patients exhibit deficiencies in one or more immunoglobulin classes, often accompanied by T-lymphocyte-predominant lymphopenia. Additionally, vaccine-specific antibody responses may be impaired due to a reduced naive B-cell pool—approximately 10% of patients present with elevated serum IgM levels. Rather than causing diagnostic confusion, this immunological profile is associated with a more progressive clinical course. It is linked to increased rates of immune dysregulation, oncologic complications, granulomatous skin lesions, and a greater predisposition to infections than others [13, 24]. In two of the cases we presented, IgM elevation was observed: Patient 1 suffered from chronic lung disease secondary to frequently recurring lower respiratory tract infections and had history of autoimmune hemolytic anaemia. Patient 3 was diagnosed with lupus vulgaris. To our knowledge, this is the first reported case of A-T diagnosed with biopsy-confirmed lupus vulgaris. This case highlights the diagnostic importance of considering atypical infections in patients with primary immunodeficiencies. In immunocompromised children, particularly those with persistent or evolving lesions, early tissue diagnosis and the inclusion of specific mycobacterial stains and cultures is essential. Timely identification of the pathogen is critical for prompt treatment and may prevent further complications in such vulnerable populations.

Although limited by its retrospective design, small sample size, and single-centre setting, the study also lacked access to extended lymphocyte subset markers (e.g., CD27, CD45RA, CD25, CD127) and functional assays such as lymphocyte proliferation in response to mitogens due to technical constraints. This limitation restricts the interpretation of the hyper-IgM phenotype and immune dysregulation, as functional assessment is crucial for distinguishing secondary IgM elevation in A–T from other primary immunodeficiencies with overlapping clinical and immunological features. Despite these limitations, the study provides valuable clinical insight by detailing the phenotypic and immunologic spectrum of pediatric A-T, particularly emphasising underrecognised presentations and real-world diagnostic complexities.

## Conclusion

This study highlights the diagnostic and clinical challenges encountered in pediatric A-T, particularly in variant cases with atypical presentations. The observed phenotypic heterogeneity and immune dysregulation underscore the importance of early and accurate diagnosis. In patients with elevated IgM or AFP levels—or in those whose clinical and laboratory findings do not fit a defined primary immunodeficiency phenotype—whole exome sequencing is essential for timely and accurate identification. Individuals with a hyper-IgM profile may experience a more severe disease course, including increased susceptibility to infections, autoimmunity, and malignancy, necessitating close monitoring. Additionally, chronic granulomatous skin lesions, which can be observed in the natural course of this disease, should be promptly evaluated to rule out atypical infections through tissue sampling. Overall, early genetic confirmation and longitudinal multidisciplinary care are critical to improving outcomes, minimising complications, and ultimately reducing the burden on affected individuals and their families.

## Data Availability

The clinical images and genetic analysis data presented in this study contain potentially identifiable patient information. Therefore, these datasets are not publicly available to ensure patient confidentiality. De-identified data may be shared upon reasonable request to the corresponding author and subject to approval by the Dokuz Eylul University Ethics Committee.

## References

[1] Gatti RA, Boder E, Vinters HV, Sparkes RS, Chen J, Albertini RJ. Localization of an ataxia-telangiectasia gene to chromosome 11q22–23. Nature. 1988;336(6199):577–80. 3200306 10.1038/336577a0

[2] Shao L, Wang H, Xu J, Qi M, Yu Z, Zhang J. Ataxiatelangiectasia in china: a case report of a novel ATM variant and literature review. Front Neurol. 2023;14:1228810. 37564729 10.3389/fneur.2023.1228810 PMC10411728

[3] Swift M, Morrell D, Cromartie E, Chamberlin AR, Skolnick MH, Bishop DT. The incidence and gene frequency of ataxia-telangiectasia in the united States. Am J Hum Genet. 1986;39(5):573–83. 3788973 PMC1684065

[4] Haskoloğlu ZŞ, Aytekin C, Bal SK, İslamoğlu C, Baskın K, Yavuz Z, et al. Does the hyper IgM phenotype affect prognosis in ataxia telangiectasia? Asthma Allergy Immunol. 2020;18(1):38–46. 10.21911/aai.523.

[5] Van Os NJH, Chessa L, Weemaes CMR, van Deuren M, Fiévet A, van Gaalen J, et al. Genotypephenotype correlations in ataxia telangiectasia patients with ATM c.3576G > A and c.8147T > C mutations. J Med Genet. 2019;56(5):308–16. 30819809 10.1136/jmedgenet-2018-105635

[6] McConville CM, Concannon P, Gatti RA, Anderson C, Martin K, Tessa A, et al. Mutations associated with variant phenotypes in ataxia-telangiectasia. Am J Hum Genet. 1996;59(2):320–30. 8755918 PMC1914715

[7] Saunders-Pullman R, Raymond D, Stoessl AJ, Hobson D, Nakamura T, Pullman S, et al. Variant ataxiatelangiectasia presenting as primaryappearing dystonia in Canadian mennonites. Neurology. 2012;78(9):649–57. 22345219 10.1212/WNL.0b013e3182494d51 PMC3286230

[8] Stankovic T, Kidd AM, Sutcliffe A, McGuire GM, Robinson P, Weber P, et al. ATM mutations and phenotypes in ataxia-telangiectasia families in the British isles: expression of mutant ATM and the risk of leukemia, lymphoma, and breast cancer. Am J Hum Genet. 1998;62(2):334–45. 9463314 10.1086/301706 PMC1376883

[9] Staples ER, McDermott EM, Reiman A, Byrd PJ, Ritchie S, Taylor AM, et al. Immunodeficiency in ataxiatelangiectasia is correlated strongly with the presence of two null mutations in the ataxiatelangiectasia mutated gene. Clin Exp Immunol. 2008;153(2):214–20. 18505428 10.1111/j.1365-2249.2008.03684.x PMC2492895

[10] European Society for Immunodeficiencies (ESID). Ataxia Telangiectasia Diagnostic Guidelines. Available from: https://esid.org/Education/Diagnostic -Criteria-PID (accessed on 6 December 2021).

[11] Nowak-Wegrzyn A, Crawford TO, Winkelstein JA, Carson KA, Lederman HM. Immunodeficiency and infections in ataxiatelangiectasia. J Pediatr. 2004;144(4):505–11. 15069401 10.1016/j.jpeds.2003.12.046

[12] Mohammadinejad P, Abolhassani H, Aghamohammadi A, Pourhamidi S, Sadeghi B, Nasiri Kalmarzi R, et al. Class switch recombination process in ataxia-telangiectasia patients with elevated serum levels of IgM. J Immunoass Immunochem. 2015;36(1):16–26. 10.1080/15321819.2014.891525 24568663

[13] Etzioni A, Ochs HD. The hyper IgM syndrome—an evolving story. Pediatr Res. 2004;56(4):519–25. 15319456 10.1203/01.PDR.0000139318.65842.4A

[14] Mizejewski GJ. Alpha-fetoprotein (AFP)-derived peptides as epitopes for hepatoma immunotherapy: a commentary. Cancer Immunol Immunother. 2009;58:159–70. 18612637 10.1007/s00262-008-0548-8 PMC11030279

[15] Mallott J, Kwan A, Church JA, Cowan MJ, Puck JM. Newborn screening for SCID identifies patients with ataxia telangiectasia. J Clin Immunol. 2013;33:540–9. 23264026 10.1007/s10875-012-9846-1 PMC3591536

[16] Richards S, Aziz N, Bale S, Bick D, Das S, Gastier-Foster J, et al. Standards and guidelines for the interpretation of sequence variants: a joint consensus recommendation of the American college of medical genetics and genomics and the association for molecular pathology. Genet Med. 2015;17(5):405–23. 25741868 10.1038/gim.2015.30 PMC4544753

[17] Marelli C, Cusano R, Bianciardi L, Brusco A, Di Gregorio E, Traversa M, et al. Mini-exome coupled to read-depth based copy number variation analysis in patients with inherited ataxias. Hum Mutat. 2016;37(12):1340–53. 27528516 10.1002/humu.23063

[18] Demuth I, Dutrannoy V, Marques W, Reis A, Sperling K, Schindler D, et al. New mutations in the ATM gene and clinical data of 25 AT patients. Neurogenetics. 2011;12:273–82. 10.1007/s10048-011-0299-0. 21965147 10.1007/s10048-011-0299-0

[19] Mitui M, Nahas SA, Du LT, Yang Z, Lai CH, Nakamura K, et al. Functional and computational assessment of missense variants in the ataxia-telangiectasia mutated (ATM) gene: mutations with increased cancer risk. Hum Mutat. 2009;30(1):12–21. 18634022 10.1002/humu.20805 PMC2776735

[20] Mutlu-Albayrak H, Kırat E, Gürbüz G. Childhood-onset autosomal recessive ataxias: a cross-sectional study from Turkey. Neurogenetics. 2020;21(1):59–66. 31741144 10.1007/s10048-019-00597-y

[21] Isik E, Onay H, Atik T, Canda E, Cogulu O, Coker M, Ozkinay F. Clinical utility of a targeted next generation sequencing panel in severe and pediatric onset Mendelian diseases. Eur J Med Genet. 2019;62(10):103725. 10.1016/j.ejmg.2019.103725. 31319225 10.1016/j.ejmg.2019.103725

[22] Micol R, Ben Slama L, Suarez F, Revuz S, Haouy S, Donadieu J, et al. Morbidity and mortality from ataxia-telangiectasia are associated with ATM genotype. J Allergy Clin Immunol. 2011;128(2):382–9. 21665257 10.1016/j.jaci.2011.03.052

[23] Szczawińska-Popłonyk A, Breborowicz A, Piotrowski A, Mikołuć B, Osmola K. Infections and immune dysregulation in ataxia-telangiectasia children with hyper-IgM and non-hyper-IgM phenotypes: A single-center experience. Front Pediatr. 2022;10:972952. 36340711 10.3389/fped.2022.972952 PMC9631935

[24] Rothblum-Oviatt C, Wright J, Lefton-Greif MA, McGrath-Morrow SA, Crawford TO, Lederman HM. Ataxia telangiectasia: a review. Orphanet J Rare Dis. 2016;11:159. 27884168 10.1186/s13023-016-0543-7 PMC5123280

[25] Liu Q, Yin Y, Wu J, Zhang M, Wang S, Huang J, et al. T cell aging as a risk factor for autoimmunity. J Autoimmun. 2023;137:102947. 36357240 10.1016/j.jaut.2022.102947 PMC10164202

[26] Kolijn PM, Langerak AW. Immune dysregulation as a leading principle for lymphoma development in diverse immunological backgrounds. Immunol Lett. 2023;263:1–7. 37774986 10.1016/j.imlet.2023.08.007

[27] Ganai S, Reshi R, Ahmad R, et al. Child with ataxia and red eyes. Postgrad Med J. 2022;98(e1):e1–2. 10.1136/postgradmedj-2021-140955 37066572

[28] Matutes E. ATM germline heterozygosity does not play a role in chronic lymphocytic leukemia initiation but influences rapid disease progression through loss of the remaining ATM allele. Leukemia. 2012;26(2):469–70. 10.3324/haematol.2011.048827 PMC3248944 21933854

[29] Blanchard-Rohner G, Moix I, Barthez M, Rougemont AL, Rivier F, Izquierdo I, et al. Childhood-onset movement disorders can mask a primary immunodeficiency: 6 cases of classical ataxia-telangiectasia and variant forms. Front Immunol. 2022;13:791522. 35154108 10.3389/fimmu.2022.791522 PMC8831727

